# Equid alphaherpesvirus 1 (EHV-1) infection of primary murine astrocytes: role of the cytoskeleton

**DOI:** 10.1007/s00705-025-06494-0

**Published:** 2025-12-11

**Authors:** Anna Słońska, Joanna Cymerys

**Affiliations:** Department of Preclinical Sciences, Institute of Veterinary Medicine, Warsaw University of Life Sciences, Ciszewskiego 8, 02-786 Warsaw, Poland

**Keywords:** Actin filaments, Equid alphaherpesvirus type 1, Microtubules, Primary murine astrocytes, Abortive infection

## Abstract

Equid alphaherpesvirus type 1 (EHV-1) is a neurotropic virus known to manipulate host cytoskeletal structures to facilitate infection and spread. In this study, we examined the susceptibility of primary murine astrocytes to neuropathogenic and non-neuropathogenic EHV-1 strains, focusing on the role of the actin and microtubule cytoskeleton. Infection induces morphological changes and remodeling of actin and microtubule networks. Viral antigen was localized along actin filaments and microtubules, indicating their likely role in intracellular viral trafficking. Cytoskeletal inhibitors significantly reduced viral DNA levels. Both EHV-1 strains exhibited limited replication, suggesting that EHV-1 may establish abortive infection in murine astrocytes.

Equid alphaherpesvirus type 1 (EHV-1) is a neurotropic herpesvirus responsible for respiratory disease, abortion, neonatal foal death, and herpes myeloencephalopathy (EHM) in horses. Although equids are its primary hosts, EHV-1 can also infect other species, including mice, which are widely used as models to study EHV-1 neuropathogenesis due to similar disease patterns and neurovirulence. In mice, EHV-1 infection leads to severe neurological disease, encephalitis, and extensive neuropathology characterized by inflammatory infiltrates and high pro-inflammatory cytokine levels in the brain [1–4].

Astrocytes, ubiquitous cells of the central nervous system (CNS), perform diverse functions, including the promotion of neuronal growth and survival, as well as the generation of innate immune responses to combat invading pathogens. Upon viral infection, astrocytes undergo significant activation, playing a pivotal role in mediating neuroinflammatory responses that can contribute to CNS pathology [5]. Productive infection of primary human astrocytes has been reported previously for several human herpesviruses (HHVs), including HHV-1 [6], HHV-2 [7], HHV-4 [8], HHV-5 [9], and HHV-6 [10, 11]. However, there is currently no definitive evidence that EHV-1 is capable of infecting these glial cells *in vitro*. Immunohistochemical studies have demonstrated the presence of EHV-1 antigen not only in neurons but also in astrocytes within the brain and spinal cord of horses exhibiting acute paralytic disease. This suggests that astrocytes, alongside neurons, can be infected directly by EHV-1 during the early stages of neuropathogenesis [12]. In murine models, EHV-1 replicates mainly in neurons of the olfactory bulb, cortex, and hippocampus, leading to CNS inflammation [1–4], although the involvement of astrocytes in these models remains to be elucidated. Unlike human herpesviruses, which clearly infect astrocytes *in vitro*, the astrocyte tropism of equine herpesviruses remains undefined. Together, current evidence suggests that astrocytes may be targets of EHV-1, potentially contributing to herpesvirus-induced neuroinflammatory damage.

EHV-1 employs multiple strategies to infect diverse hosts and cell types, including manipulation of the host cytoskeleton [13–16]. Herpesviruses can modulate the cytoskeleton at nearly every step of their replication cycle, from entry to egress, to facilitate their spread. While cytoskeletal remodeling is a hallmark of alphaherpesviruses, the specific mechanisms and dependencies vary significantly across this subfamily. For example, cytoskeletal elements may serve as directional tracks for viral transport or be actively disrupted when posing a physical barrier [17, 18]. Additionally, herpesviruses have evolved mechanisms to evade the host immune system by transmitting progeny virions directly to neighboring permissive cells via cell junctions, thus bypassing extracellular release [19].

In prior work, we demonstrated that EHV-1 critically relies on microfilaments (actin) and microtubules during infection of primary murine neurons [13, 14], the glioblastoma multiforme cell line A172 [16], equine dermal cells (ED), and Vero cells [15]. Here, we extend these findings by investigating whether neuropathogenic and non-neuropathogenic EHV-1 strains differ in their ability to infect primary murine astrocytes *in vitro* and by defining the role of the host cytoskeleton during the infection process.

Primary astrocyte cultures were prepared from 7- to 10-week-old BALB/c (H-2d) mice, following a previously described protocol [7], with animal care in compliance with Polish and EU regulations. Cells were grown in glial growth medium (DMEM with GlutaMAX™, 0.6% glucose, 1% penicillin/streptomycin, and 10% fetal bovine serum [FBS]) (Life Technologies) and seeded on poly-D-lysine/laminin-coated slides (10^5^ cells/well). Astrocyte purity was ensured following the protocol of Güler et al. [20]. The purity of the astrocyte cultures exceeded 99%, as confirmed by GFAP immunostaining.

Two EHV-1 strains from the virus collection of the Virology Laboratory at Warsaw University of Life Sciences-SGGW were used: (i) the non-neuropathogenic strain Jan-E, which was confirmed to lack neuropathogenicity using a PCR-RFLP neuropathogenic/non-neuropathogenic discrimination test [21], and (ii) neuropathogenic strain of EHV-1 (EHV-1 26), which was isolated in Hungary in 2004 and the neuropathogenicity of which was confirmed using the PriProET technique [22]. Primary murine astrocytes were infected at an MOI of 1.0 for 60 min at 37°C. After removing the inoculum and washing with PBS, cells were incubated for 2, 24, and 48 hours at 37°C with 5% CO_2_ in fresh growth medium.

For immunofluorescence, astrocytes were cultured on coverslips in 6-well plates and infected with EHV-1 strains. Cells were fixed by adding 4% paraformaldehyde, permeabilized with 0.5% Triton X-100 in PBS, and blocked with 1% bovine serum albumin in PBS (Sigma-Aldrich). GFAP and microtubules were stained using mouse monoclonal antibodies – anti-GFAP (Calbiochem, 1:200, 60 min, 37°C) and anti-α-tubulin (Thermo Fisher, 1:100, 60 min, 37°C), respectively – followed by Texas Red-X–conjugated secondary antibodies (Invitrogen, 1:1000, 60 min, RT) for visualisation. Viral antigens were detected by direct immunofluorescence using an anti-equine rhinopneumonitis virus/equine herpesvirus type I (ERV/EHV-1) polyclonal antiserum conjugated to FITC (VMRD, Inc.). The F-actin was labeled with TRITC-phalloidin (Sigma Chemicals, 500 ng/ml, 60 min), and nuclei were stained with Bisbenzimidine/Hoechst 33258 (Sigma Aldrich, 2 µg/ml). Control cells were treated identically but not infected. Slides were mounted in ProLong Gold Antifade Reagent (Invitrogen) and imaged using a Fluoview FV10i confocal microscope (Olympus). Images were processed with ImageJ (NIH Image, version 1.53q, USA) and Adobe Photoshop CS6 software (Adobe Systems Incorporated).

Primary murine astrocytes (4 × 10^5^ cells) in 12-well plates were either mock-treated or pre-treated for 1 hour at 37°C with cytoskeletal inhibitors – latrunculin A (0.5, 1 µM), cytochalasin D (4, 6, 10 µM), and nocodazole (15, 30, 45 µM) – based on previously validated MTT assays [13, 14]. Following drug treatment, the cells were infected with EHV-1 at an MOI of 1.0. After 1 hour of incubation, the inoculum was removed, fresh culture medium was added, and the cells were further incubated for 2 and 24 hours. Viral DNA was extracted from the cell lysates and supernatants using a High Pure Viral Nucleic Acid Kit (Roche Diagnostics) and stored at -20°C for subsequent quantitative real-time PCR analysis.

EHV-1 DNA was quantified by real-time PCR (qPCR) using TaqMan probes (150 nM) targeting the EHV-1 gB gene. The sequences of the primers and probe were as follows: forward, 5’CACGTCTTTAGCGGTGAT3’; reverse, 5’CAAGCTCGTTCAGGTACAG 3’; probe, FAMTGCATTCAGACCTATGCTCTCCAAC-BHQ. The reaction mixture was prepared using a TaqMan Master Kit (Roche Diagnostics) and run on a LightCycler 2.0 instrument (Roche Diagnostics) according to a previously established protocol [13, 14]. Fluorescence levels were measured at a wavelength of 530 nm, which is specific for the FAM fluorophore dye.

Statistical analysis was performed using one-way ANOVA with Tukey’s multiple comparisons test in GraphPad InStat v3 (GraphPad Software Inc., San Diego, CA, USA). Data are presented as the mean ± SEM from at least three independent experiments. Statistical differences were defined as significant at *p* ≤ 0.05 (*), highly significant at *p* ≤ 0.01 (**), extremely significant at *p* ≤ 0.001 (***), and not significant at *p* > 0.05 (ns).

We evaluated the susceptibility of primary murine astrocytes to two EHV-1 strains: the non-neuropathogenic strain Jan-E and the neuropathogenic strain EHV-1 26. Dual immunofluorescence and confocal microscopy revealed that viral antigen colocalized with GFAP, a cytoskeletal protein specific to astrocytes. GFAP maintains astrocyte morphology, supports the cytoskeletal structure, and mediates cell migration and intercellular communication [23, 24]. In mock-infected astrocytes, GFAP formed a dense, radial network (Fig. 1A), while EHV-1 infection disrupted this organization and induced morphological changes. By 24 and 48 h postinfection (h p.i.), astrocytes often lost their stellate shape, becoming hypertrophic and rounded, with retracted processes (Fig. 1B and C, white squares). These GFAP disruptions and morphological changes are consistent with prior observations in herpesvirus-infected astrocytes [7, 25]. Moreover, viral antigen localized mainly in the perinuclear cytoplasm (Fig. 1B and C, white arrows) and at 48 h p.i. was also detected within GFAP-positive projections (Fig. 1B and C, yellow arrows). Similar process-associated structures were observed previously in HHV-2-infected astrocytes, suggesting a potential role in direct cell-to-cell viral spread [7].

**Fig. 1:**
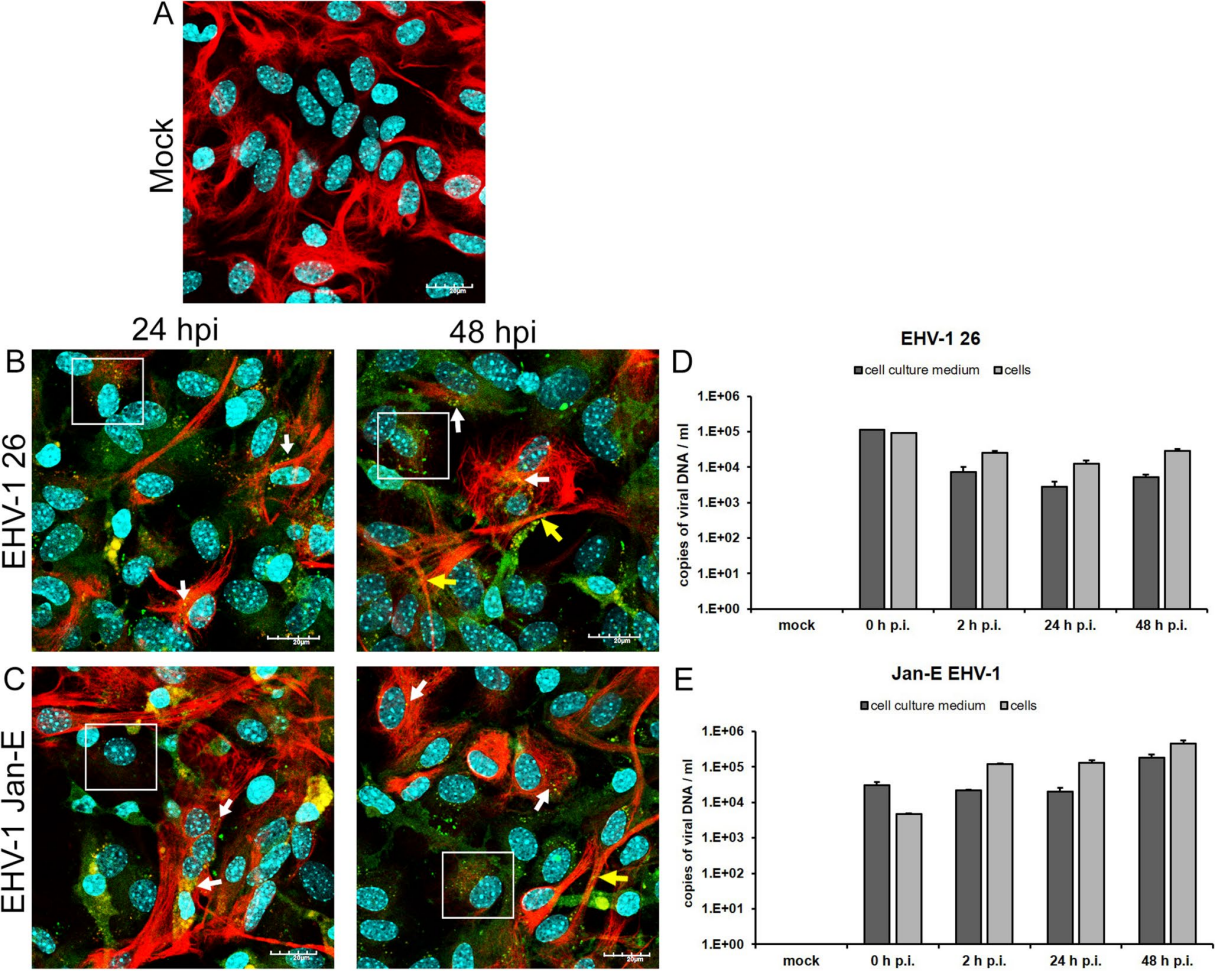
Representative confocal images of mock-infected (**A**), EHV-1 26-infected (**B**), and Jan-E-infected (**C**) primary murine astrocytes at 24 and 48 h p.i. The maximum projection of the combined confocal images shows EHV-1 antigen (green), GFAP (red), and cell nuclei (blue). White arrows indicate perinuclear antigen accumulation; yellow arrows point to antigen in GFAP-positive projections. White boxes highlight infected astrocytes with a globoid morphology and retracted processes. Magnification, 60×; scale bars, 20 µm. Panels D and E show qPCR-based viral DNA levels at 48 h p.i.. Data are the mean ± SEM (*n* = 3)

qPCR analysis revealed distinct replication dynamics between the two EHV-1 strains in infected astrocytes. In cells infected with EHV-1 strain 26, viral DNA copy numbers changed markedly over time. At 0 h p.i. (after a 1-hour adsorption), both the culture medium and cells contained approximately 1.0 × 10^5^ copies/ml. By 2 h p.i., a decrease of about one log_10_ unit was observed in the medium (~ 1.0 × 10^4^ copies/ml), while a less pronounced reduction occurred in the cells (~ 3.0 × 10^4^ copies/ml). At 24 h p.i., viral DNA levels declined further to ~ 3.0 × 10^3^ in the medium and ~ 1.0 × 10^4^ in the cells, representing a reduction of 1–2 log_10_ units relative to the initial value. By 48 h p.i., a slight increase was detected, with ~ 1.0 × 10^4^ and ~ 3.0 × 10^4^ copies/ml in the medium and cells, respectively, indicating a partial increase in viral DNA levels (Fig. 1D). Astrocytes infected with EHV-1 strain Jan-E displayed a distinct replication profile. At 0 h p.i., ~ 1.5 × 10^4^ copies/ml were detected in the medium and ~ 6.0 × 10^3^ in the cells. By 2 h p.i., the viral load in the medium remained stable (~ 1.5 × 10^4^ copies/ml), whereas in the cells it increased by approximately one log_10_ unit to ~ 1.5 × 10^5^ copies/ml. At 24 and 48 h p.i., viral DNA levels remained high in the cells (~ 1.5 × 10^5^-10^6^ copies/ml) and increased moderately in the medium (up to ~ 1.0 × 10^5^ copies/ml) (Fig. 1E).

These findings suggest that a population of EHV-1-infected primary murine astrocytes is unable to complete productive infection. This may reflect an abortive infection, which refers to a state where the virus infects a cell but fails to produce progeny virus, typically due to the nonpermissive nature of the cellular environment. Recent studies have shown that abortive infection by herpesviruses, particularly HHV-1, occurs not only in nonpermissive cells but also in permissive non-neuronal cells. In such cases, viral genomes persist in a quiescent, non-replicating form within the nucleus for extended periods without inducing cytopathic effects while retaining the capacity for spontaneous reactivation and production of infectious virus under certain conditions [26]. The results demonstrate that viral DNA was predominantly cell-associated, with significantly lower levels detected in the supernatant, further supporting the conclusion that little to no infectious progeny was released. It is therefore plausible that EHV-1 establishes a similar abortive infection in astrocytes. However, this hypothesis requires further investigation, particularly through studies focusing on long-term viral persistence and reactivation dynamics.

EHV-1 infection induced several changes in the distribution of actin filaments within primary murine astrocytes. In mock-infected cells, actin filaments formed stress fibers beneath the cortex, with prominent structures such as tunneling nanotubes (TNTs) and filopodia (Fig. 2A). Cytoskeletal remodeling was evident as early as 2 h p.i., marked by a reduction and thinning of stress fibers in the perinuclear region (Fig. 2B and E, asterisks), a pattern also seen at 24 h p.i. (Fig. 2C and F, asterisks). At both time points, viral antigen accumulated in the perinuclear area, the site of viral genome replication (Fig. 2B, C, E, and F, white arrows), and localized along thin actin filaments (Fig. 2B and E, magnified area, white arrowheads), suggesting the involvement of actin in viral trafficking. At the early stage of infection (2 h p.i.), the antigen along actin fibers likely represented incoming virions being transported toward the nucleus. By 24 h p.i., viral antigen was associated with filopodia, implying a potential role in cell-to-cell spread of progeny virions (Fig. 2C and F, magnified areas, arrowheads). Colocalization analysis using BIOP JACoP revealed a moderate correlation between viral antigen and F-actin (Pearson’s r = 0.4–0.55 for both strains). Manders’ M2 coefficient (~ 0.9) indicated that most viral antigen overlapped with F-actin, while M1 values (0.6–0.7 for EHV-1 26 and 0.5–0.6 for Jan-E EHV-1) showed that only a portion of the actin filaments colocalized with the antigen (Fig. 2D and G).

**Fig. 2:**
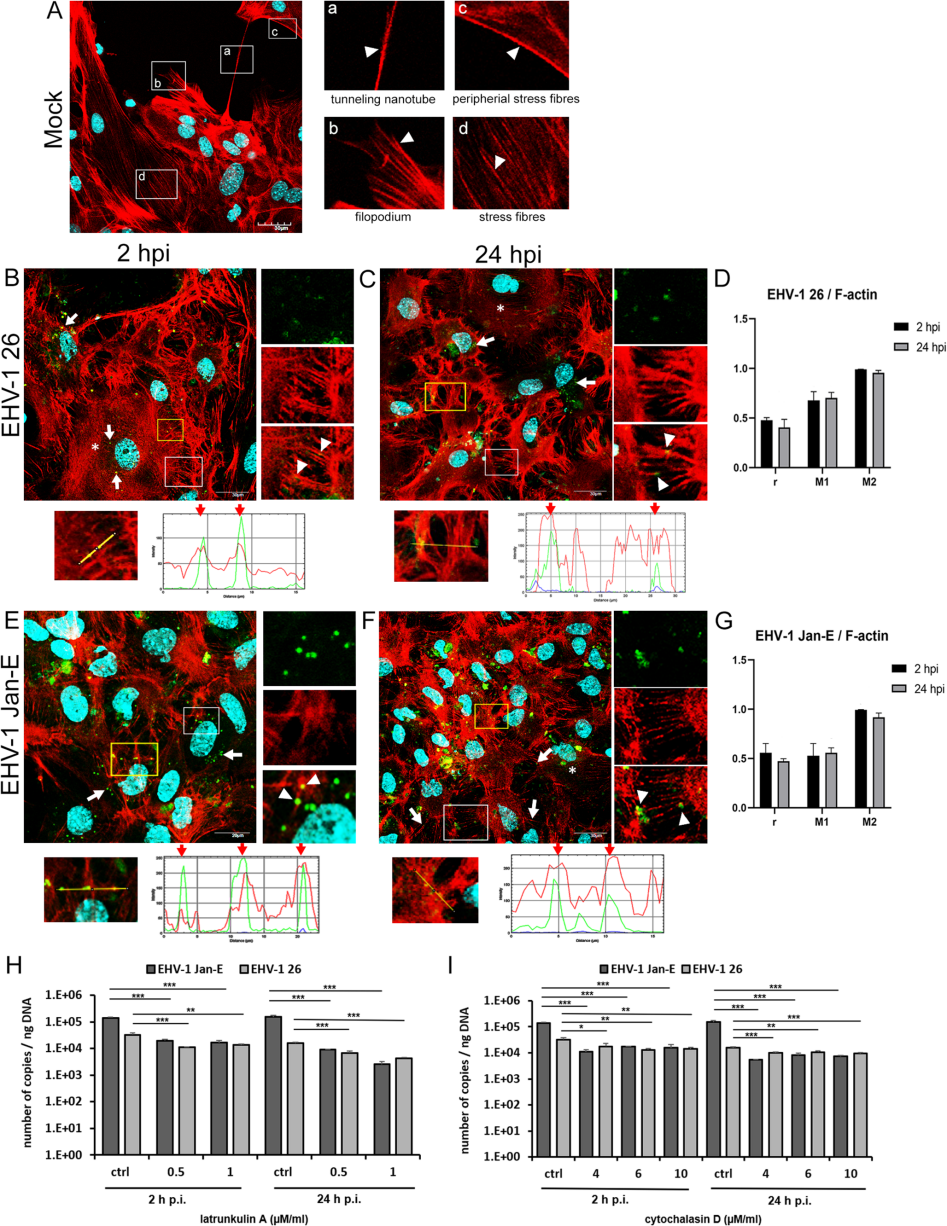
Representative confocal images of mock-infected (**A**), EHV-1-26-infected (**B**, **C**), and Jan-E-infected (**E**, **F**) astrocytes at 2 and 24 h p.i. The maximum projection of the combined confocal images shows EHV-1 antigen (green), F-actin (red), and cell nuclei (blue). Panel A displays typical actin structures in uninfected cells (white boxes a-d). In B-C and E-F, white arrows indicate viral antigen along actin filaments, white arrowheads indicate colocalization sites in magnified ROIs, and asterisks indicate reduced and thinned stress fibers. Scale bars: 20 or 30 µm. Line profile plots display green/red fluorescence intensity along yellow ROI lines, with red arrows marking overlap zones. Panels D-G: colocalization analysis (Pearson’s r, Manders’ M1/M2) in ≥ 50 cells. Panels H-I: qPCR quantification of viral DNA at 2 and 24 h p.i. following actin disruption (latrunculin A, cytochalasin D). Data are presented as the mean ± SEM (*n* = 3); *, *p* ≤ 0.05; **, *p* ≤ 0.01; ***, *p* ≤ 0.001 (Tukey’s test)

Regarding microtubule structure, mock-infected astrocytes showed a continuous network of microtubules radiating from the nucleus to the cell periphery (Fig. 3A). At 2 h p.i., no major structural changes were observed, although viral antigens localized along tubulin fibers, mainly at the cell periphery (Fig. 3B and E, white arrows). By 24 h p.i., the microtubule network was disrupted, particularly in astrocytes infected with the EHV-1 Jan-E strain, which caused marked thinning and depolymerization of microtubules (Fig. 3F, asterisks). Infection with the EHV-1 26 strain also led to rarefaction of peripheral microtubules, while a dense α-tubulin network persisted around the nucleus, where viral antigen accumulated. Pearson’s correlation coefficients (EHV-1 26: r = 0.541 and 0.712; Jan-E: r = 0.679 and 0.850 at 2 and 24 h p.i., respectively) indicated a strong positive correlation between α-tubulin and viral antigen. Manders’ M1 and M2 coefficients (0.7-1.0) further confirmed co-localization between α-tubulin and the viral antigen (Fig. 3D and G).

**Fig. 3:**
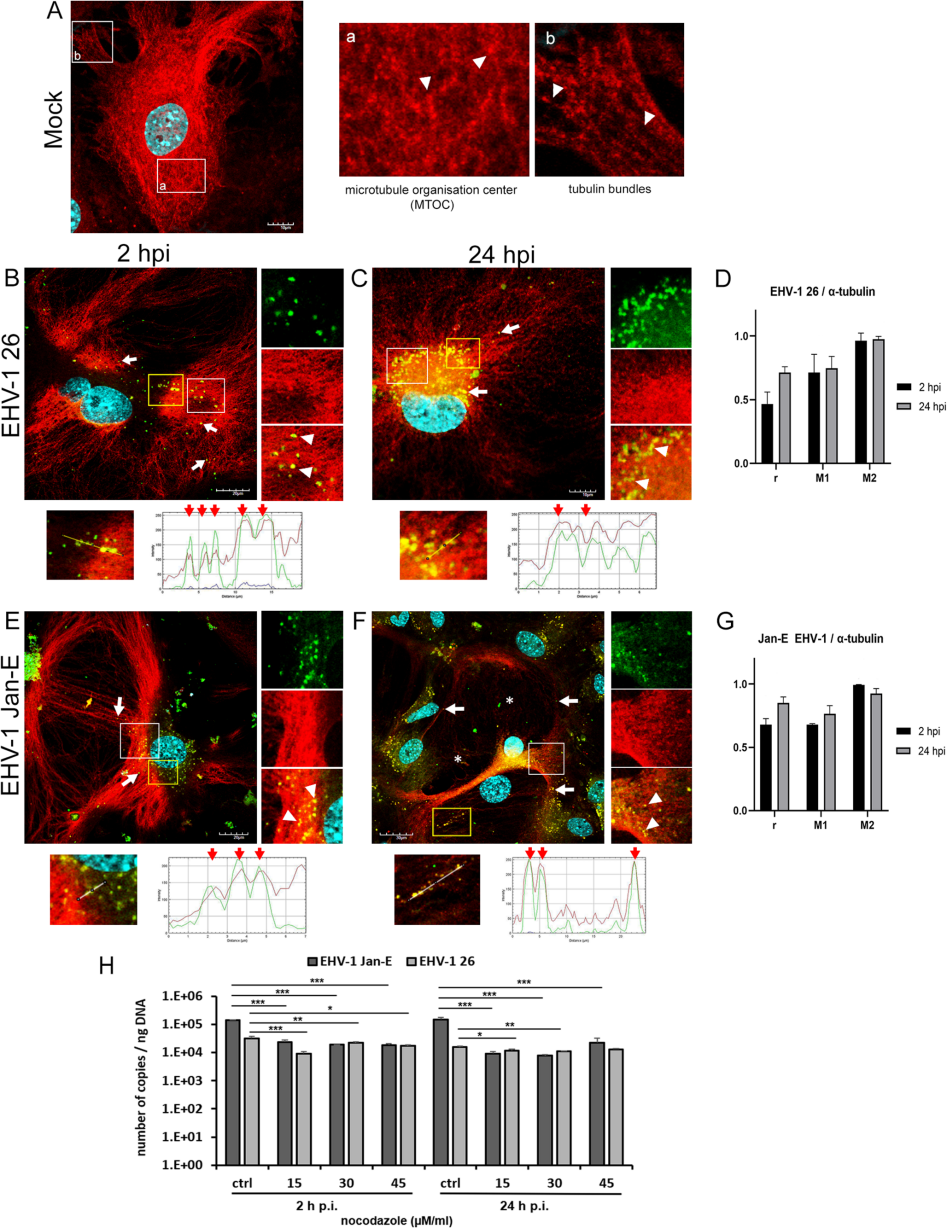
Representative confocal images of mock-infected (**A**), EHV-1-26-infected (**B**, **C**), and Jan-E-infected (E, F) astrocytes at 2 and 24 h p.i. The maximum projection of the combined confocal images shows EHV-1 antigen (green), β-tubulin (red), and cell nuclei (blue). Panel A shows the typical microtubule network in control cells (white boxes a-b). In B-F, white arrows indicate viral antigen aligned with microtubules; arrowheads indicate colocalization in magnified ROIs. Scale bars: 10–30 µm. Line profile plots display green/red fluorescence intensity along yellow ROI lines, with red arrows marking overlap zones. Panels D and G present colocalization analysis of EHV-1 antigen and β-tubulin using Pearson’s r and Manders’ M1/M2 (≥ 50 cells). Panel H shows qPCR-based viral DNA quantification at 2 and 24 h p.i. in cells pre-treated with nocodazole and infected with Jan-E or EHV-1 26. Data are presented as the mean ± SEM (*n* = 3); *, *p* ≤ 0.05; **, *p* ≤ 0.01; ***, *p* ≤ 0.001 (Tukey’s test)

To investigate the role of the cytoskeleton in EHV-1 infection, primary murine astrocytes were pre-treated with cytochalasin D or latrunculin A (actin depolymerizers) or nocodazole (a microtubule-disrupting agent), followed by infection with either the Jan-E or 26 EHV-1 strain. qPCR analysis at 2 and 24 h p.i. revealed a significant, concentration-dependent reduction in viral DNA copy number for both strains (Figs. 2H and I and 3H). Actin-targeting agents effectively suppressed viral replication: cytochalasin D reduced EHV-1 26 DNA levels by ~ 0.3 log_10_ at 2 h p.i. and ~ 0.2 log_10_ at 24 h p.i., while latrunculin A caused a ~ 0.4- to 0.6-log_10_ reduction. The Jan-E strain was more sensitive, with cytochalasin D reducing viral DNA levels by ~ 0.9–1.1 log_10_ (2 h p.i.) and ~ 1.3–1.5 log_10_ (24 h p.i.), and latrunculin A achieving the strongest effect (~ 1.2–1.8 log_10_). Nocodazole also inhibited replication, reducing Jan-E DNA levels by ~ 0.8–0.89 log_10_ (2 h p.i.) and ~ 1.18–1.26 log_10_ (24 h p.i.), while EHV-1 26 showed smaller reductions (~ 0.17–0.28 log_10_ at 2 h p.i.; ~0.08–0.16 log₁₀ at 24 h p.i.).

Although no productive infection was observed, infected astrocytes exhibited distinct morphological alterations, including cytoskeletal remodeling. In EHV-1-infected astrocytes, actin-based filopodia and cellular protrusions were observed, along which the viral antigen was detected, suggesting their involvement in both intra- and extracellular viral transport. Numerous studies have demonstrated that various actin-rich extensions are utilized by herpesviruses, including EHV-1, bovine herpesvirus type 1 (BoHV-1), pseudorabies virus (PRV), and HHV-1, not only prior to infection – to facilitate 'surfing' toward entry sites – but also after replication, allowing virions to reach adjacent uninfected cells [13, 19, 27, 28]. These findings confirm that actin-mediated transport represents a potential target for future studies aimed at better understanding virus-host interactions and developing antiviral strategies that interfere with cytoskeletal dynamics.

Microtubule-dependent intracellular transport is also exploited by herpesviruses, including EHV-1, during infection of various cells. Incoming capsids are usually transported along the microtubule network toward the nucleus, where viral replication, transcription, and capsid assembly occur [14, 29]. Confocal microscopy analysis revealed that EHV-1 antigen was located along the α-tubulin fibers, particularly in the perinuclear region, suggesting that the microtubule system facilitates the delivery of EHV-1 genomes to the nucleus. Moreover, disruption of both actin and microtubule networks using cytoskeleton inhibitors hinders early stages of infection and reduces viral replication, with the Jan-E strain showing greater sensitivity to cytoskeletal interference than the neuropathogenic EHV-1 26.

In conclusion, this study demonstrates that EHV-1 can infect primary murine astrocytes *in vitro*. Both neuropathogenic and non-neuropathogenic strains induced significant morphological changes in astrocytes, disrupting the organization of actin filaments and microtubules. The colocalization of viral antigens with cytoskeletal components suggests their role in viral trafficking. Furthermore, disrupting cytoskeletal structures significantly reduced viral DNA levels, underscoring the importance of the cytoskeleton in early stages of EHV-1 infection. Although the infection does not result in complete viral replication, indicating the establishment of an abortive infection, it nevertheless induces significant morphological changes and cytoskeletal remodeling. These findings expand our understanding of EHV-1 tropism and cytoskeletal interactions in non-neuronal brain cells, underscoring the need for further studies on the potential contribution of astrocyte infection to neuroinflammation and viral persistence *in vivo*.

## Data Availability

The data that support the findings of this study are available from the corresponding author upon reasonable request.
